# Consumption of Sylimarin, Pyrroloquinoline Quinone Sodium Salt and Myricetin: Effects on Alcohol Levels and Markers of Oxidative Stress—A Pilot Study

**DOI:** 10.3390/nu16172965

**Published:** 2024-09-03

**Authors:** Gerardo Bosco, Alessandra Vezzoli, Andrea Brizzolari, Matteo Paganini, Tommaso Antonio Giacon, Fabio Savini, Maristella Gussoni, Michela Montorsi, Cinzia Dellanoce, Simona Mrakic-Sposta

**Affiliations:** 1 Department of Biomedical Sciences, University of Padua, 35122 Padua, Italy; gerardo.bosco@unipd.it (G.B.); andrea.brizzolari@unipd.it (A.B.); tommasoantonio.giacon@studenti.unipd.it (T.A.G.); 2 Institute of Clinical Physiology, National Research Council (CNR), 20159 Milan, Italy; maristella.gussoni@unimi.it (M.G.); michela.montorsi@uniroma5.it (M.M.); cinziacarla.dellanoce@cnr.it (C.D.); simona.mrakicsposta@cnr.it (S.M.-S.); 3 Pharmatoxicology Laboratory—Hospital “Santo Spirito”, 65100 Pescara, Italy; fabio.savini@asl.pe.it

**Keywords:** alcohol level, ethyl glucuronide, ROS, biomarkers, oxidative stress

## Abstract

Background: Alcohol abuse is one of the most common causes of mortality worldwide. This study aimed to investigate the efficacy of a treatment in reducing circulating ethanol and oxidative stress biomarkers. Methods: Twenty wine-drinking subjects were investigated in a randomized controlled, single-blind trial (ClinicalTrials.gov. Identifier: NCT06548503; Ethical Committee of the University of Padova (HEC-DSB/12-2023) to evaluate the effect of the intake of a product containing silymarin, pyrroloquinoline quinone sodium salt, and myricetin (referred to as Si.Pi.Mi. for this project) on blood alcohol, ethyl glucuronide (EtG: marker for alcohol consumption) and markers of oxidative stress levels (Reactive Oxygen Species—ROS, Total Antioxidant Capacity—TAC, CoQ10, thiols redox status, 8-isoprostane, NO metabolites, neopterin, and uric acid). The effects of the treatment versus placebo were evaluated acutely and after 1 week of supplementation in blood and/or saliva and urine samples. Results: Si.Pi.Mi intake reduced circulating ethanol after 120 min (−33%). Changes in oxidative stress biomarkers, particularly a TAC (range +9–12%) increase and an 8-isoprostane (marker of lipidic peroxidation) decrease (range −22–27%), were observed too. Conclusion: After the administration of Si.Pi.Mi, the data seemed to suggest a better alcohol metabolism and oxidative balance in response to wine intake. Further verification is requested.

## 1. Introduction

Alcohol abuse is one of the most common causes of mortality worldwide, due to its deleterious effects on the liver, brain, heart and gut [1,2]. Ethanol can be metabolized by a healthy liver at the rate of about 8 g per hour. Liver functions are impaired by diseases such as hepatitis, cirrhosis, gallbladder and cancer, or by excessive ethanol consumption, leading to a slower rate of ethanol metabolism [3]. Ethanol is processed by alcohol dehydrogenase (ADH), which is found in several tissues including the gastric mucosa, into acetaldehyde and further into acetic acid by acetaldehyde dehydrogenases (ALDH) in hepatic mitochondria, releasing acetyl-CoA. Once acetyl-CoA is formed, it becomes a substrate for the citric acid cycle, ultimately producing cellular energy and releasing water and carbon dioxide. Excessive ethanol consumption leads to an increase in reactive oxygen species (ROS) such as 1-hydroxyethyl radical (HER), superoxide anion and hydroxyl radical [4], reducing the endogenous detoxifying mechanism efficacy, i.e., the oxidative metabolism of monooxygenase, which includes cytochrome P450 [5]. Superoxide anion may combine with nitric oxide (NO), giving rise to aggressive reactive nitrogen species (RNS) such as peroxynitrite (ONOO-) that, besides inactivating some NO synthases [6], lead to biological macromolecule damage [7]. Circulating ROS increase is also promoted by an excessive mobilization of ferritin and free iron through the Fenton reaction [8]. Alcohol abuse impairs endogenous antioxidant defenses, reducing retinol, α-tocopherol and glutathione concentrations [9]. Vitamin E deficiency promotes lipid peroxidation (LPO), exacerbating malondialdehyde (MDA) production [10]. A lack of retinol contributes to lysosomal injury while a glutathione concentration decrease leads to mitochondrial dysfunction, as well as to a greater cell apoptosis susceptibility [11]. 

In fact, there are no substances that can decrease the blood alcohol level and/or reduce, in less than physiological time, the alcohol concentration of blood: 12 g of alcohol corresponds to 120 mL of wine (12%), 300 mL of beer (5%) or 30 mL of spirits (40%) [12]. This ethanol amount is completely metabolized in 2–3 h. To get the blood alcohol level back to zero, the human body needs at least 2–3 h for each drink, depending on several factors including a person’s weight, gender and whether they have an empty/full stomach [13]. There are several products that claim to reduce/control the blood alcohol level, but their effectiveness is not supported by any scientific evidence [14].

The only data available are based on animal models where the administration of some plant extracts (rich of polyphenols) may promote the activity of antioxidant enzymes such as superoxide dismutase (SOD) and decrease circulating MDA [15]. Products containing silymarin, a mixture of silibin, silicristin, and silidianin [16], in a 3:1:1 ratio respectively, have been tested on mice; these have shown an immediate detoxifying action, reducing, in the short term, the blood alcohol content [17]. Silibin, silicristin, and silidianin are active ingredients found in several plants, including milk thistle. Myricetin, extracted from *Myrica cerifera* and tested on animal models, has also been shown to reduce ethanol in the bloodstream by activating ADH to catabolize circulating alcohol [18]. More recently, pyrroloquinoline quinone sodium salt (PQQ) has been approved as a novel food by the European Food Safety Authority (EFSA): tested on an animal model, it promotes ADH, catalase (CAT) and SOD activity, leading to a reduction in oxidative stress [19]. Some authors have investigated the effects of red wine intake on blood alcohol levels [20]: the results are controversial because the higher blood alcohol in women than in men may be due to different body compositions, gender differences and gastric ADH, reduced in women [21]. 

The aim of this study was to evaluate, against a control group, the blood alcohol and urinary ethyl glucuronide (marker for alcohol consumption) concentrations and the changes in oxidative stress biomarkers (ROS, Total Antioxidant Capacity-TAC, CoQ10, thiols redox status, 8-isoprostane, NO metabolites, neopterin, and uric acid) after the consumption of a product devised to reduce circulating ethanol.

## 2. Materials and Methods

### 2.1. Subjects

This study was a randomized, patient-blinded, controlled trial (ClinicalTrials.gov, https://clinicaltrials.gov/search?cond=%20NCT06548503 (accessed on 9 August 2024) Identifier: NCT06548503), performed at the Physiology Laboratories of the University of Padova (Padova, Italy); it was conducted in accordance with the Good Clinical Practice guidelines and the Declaration of Helsinki [22]. Study approval was received from the Ethical Committee of the University of Padova (HEC-DSB/12-2023).

Twenty wine-drinking white subjects, 13 men and 7 women, (age: 21.40 ± 2.46 years; height: 167.30 ± 3.62 cm; weight: 60.11 ± 10.53 kg; BMI: 21.36 ± 2.82 kg·m^−^^2^), were investigated in a randomized controlled, single-blind trial to evaluate the intake of a product containing silymarin, pyrroloquinoline quinone sodium salt, and myricetin (referred to as Si.Pi.Mi. for this project) on alcohol levels and markers of oxidative stress.

The inclusion criteria were as follows:

- aged between 18 and 35 years;
- no history of alcohol abuse or other substances;
- white ethnicity;
- non-smoker;
- good health condition, with no autoimmune, endocrine, infectious, cardiac, renal, hepatic or metabolic diseases;
- no pregnancy or lactation conditions.

Subjects were asked to avoid the use of aspirin, paracetamol, or other anti-inflammatory drugs in the 7 days before and during the experiment, and avoid the consumption of caffeine in the 12 h before the test. Furthermore, volunteers were asked to avoid intaking any alcohol in the 7 days before and during the experiment, except the one consumed for the experiment. 

Furthermore, before starting the study, the participants’ wine drinking habits were assessed by the following question: “how much wine do you consume per day?” “0 glasses, 1–3 glasses, or more than 3?”. About 50% of the recruited subjects belonging to both groups (treated vs. placebo) said that they consumed 1 glass of wine per day; the others declared that they did not drink alcohol.

All subjects were informed about the risks and benefits of this study, read and signed a specific informed consent form before the experiment, and gave personal anthropometric data.

Venous blood samples were drawn for the standardized clinical hematological analyses. Parameters: the mean cell volume (MCV) and hepatic function parameters including aspartate transferase (AST), alanine transferase (ALT), γ-glutamyltransferase (γ GT), total and fractionated bilirubin were determined by using an automated hematology analyzer (cobas®, Basel, Switzereland), according to the standard analysis methods. 

### 2.2. Experimental Protocol

After inclusion in the study, the subjects were randomly divided, by research staff not involved in the trial and using an electronic number generator (https://www.calculator.net/random-number-generator.html, accessed on 9 August 2024), into the following two groups:

(A) Placebo (sham control) group.
(B) Si.Pi.Mi group.

No subject knew what group he/she belonged to (“placebo” or “Si.Pi.Mi”).

The Si.Pi.Mi. group was supplemented with a commercial complex (AGfit3, Alchimia Innovazione srl, Padova, Italy). AGfit3 contains active agents coming from two plants: Silymarin and *Myrica cerifera*, traditionally used for treating the liver, combined with the sodium salt of Bio-PQQ pyrroloquinoline quinone. AGfit3 was added to an aqueous solution of orange granular, freeze-dried orange, citric acid and sucrose. The placebo adopted was an aqueous solution of ascorbic acid (1 g), orange granular, freeze-dried orange, citric acid and sucrose. The placebo and Si.Pi.Mi. were endowed with a similar color and flavor.

**Figure 1:**
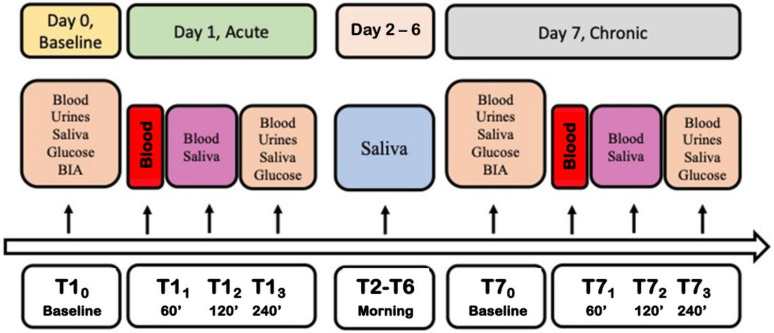
Experiment design and timeline. BIA: Bioelectrical impedance analysis.

In this experiment, all subjects were asked to drink a glass (150 mL) of Cabernet red wine (Cantine Ca’ Lustra Zanovello, Cinto Euganeo, PD, Italia) with an alcohol content of 12.5 ± 0.5%, corresponding to 14.81–15.40 g of ethanol according to the drink’s average dose reported by Kerr et al. [23]. The experiment was carried out as reported in Figure 1.

Biological samples were collected, and measurements were carried out as follows:

- Day 0: baseline before the experiment. Blood, saliva and urine samples were collected (basal measures).
- Day 1: to evaluate the acute wine intake, blood samples were drawn 60, 120 and 240 min after drinking (150 mL of red wine) to establish an ethanol metabolism curve. The placebo or Si.Pi.Mi. were taken after the wine dose. Saliva was collected 120 and 240 min after drinking, while urine was collected after 240 min.
- Day 2–6: for the long-term intake, volunteers drank one wine glass at lunch and another one at dinner (300 mL/die, corresponding to 30 g of ethanol/die approximately). They consumed the wine dose at the beginning of the meal, and then continued to eat a balanced daily menu according to individual energy needs. They took the placebo or Si.Pi.Mi. after the meal, depending on the group. Saliva was collected in the morning.
- Day 7: biological samples were collected in the same way as day 1, up to the end of the study.

### 2.3. Blood, Saliva and Urine Samples

After enrollment, for each subject, in the morning before breakfast, venous blood samples (about 5 mL) were drowned in EDTA and LH tubes (Vacuette tube, Greiner bio-one, Kremsmünster, Austria). The blood samples were centrifugated (Hettich^®^GmbH & Co. KG, MIKRO 200R centrifuge, Tuttlingen, Germany) for 10 min to separate plasma and red blood cells (RBC). Multiple aliquots were immediately frozen and stored at −80 °C. Plasma samples were collected to determine the blood ethanol, ROS, TAC, and Co Q10 levels. Saliva samples were collected to determine the ROS and TAC, while urine samples were collected to determine the Ethyl Glucuronoide (ETG), lipid peroxidation (8-iso-PGF2α), NO metabolites (NOx), and creatinine, neopterin, and uric acid concentration. Aminothiols’ redox status was evaluated by an RBC analysis.

### 2.4. Biomarker Analysis

#### 2.4.1. Blood Alcohol Level

The blood ethanol level was determined as previously described [24] using an Agilent 7820 A series GC instrument, and HP-Innowax (30 m × 0.25 mm × 0.25 μm) capillary column (Agilent, Santa Clara, CA, USA). Helium (flow = 1.5 mL/min) was used as the carrier gas. The injector, the column and the detector were maintained at 250 °C, 40 °C and 250 °C, respectively. The analysis was carried out by isothermal elution. *n*-Propanol was used as the internal standard. Nitrogen, hydrogen and air were used as gas for the FID detector at 25, 40 and 400 mL/min, respectively.

#### 2.4.2. Urine Ethyl Glucuronoide (ETG)

ETG is a metabolic product of ethylic alcohol coming from the reaction of ethanol and acid glucuronic. ETG was determined in urine samples using a commercial enzymatic immunoassay (quantILAB^®^ n° W1510011723, Ascoli Piceno, Italy) according to the manufacturer’s instructions. All measures were assessed in duplicate. The inter-assay coefficient of variation was in the range indicated by the manufacturer.

#### 2.4.3. Plasma ROS Production 

An X-band Electron Paramagnetic Resonance Spectroscopy (EPR) instrument (E-Scan, 9.3 GHz Bruker Co., Billerica, MA, USA) was used to detect ROS production in the plasma and saliva samples, following previously described methods [25,26,27]; using the spin probe CMH (1-hydroxy-3-methoxy-carbonyl-2,2,5,5-tetramethylpyrrolidine), the spectra could be acquired and the ROS resonances detected. The stable radical CP (3-carboxy2,2,5,5-tetramethyl1-1-pyrrolidi-nyloxy) was then adopted as an external reference to convert the ROS calculated data into absolute quantitative levels (µmol·min**^−^**^1^).

#### 2.4.4. Total Antioxidant Capacity (TAC)

The 6-hydroxy-2,5,7,8-tetramethylchroman-2-carboxylic acid (Trolox-) equivalent antioxidant capacity assay, a widely used kit-based commercial method was used (Cayman Chemical, Ann Arbor, MI, USA, Item No. 709001) [26,28]. Briefly, 10 μL of plasma and/or saliva was added in duplicate to 10 μL of metmyoglobin and then 150 μL of the chromogen solution; the reactions were started by the addition of 40 μL of hydrogen peroxide. The reaction mixtures were incubated for 3 min at room temperature; then, the absorbance signal at 750 nm was determined by an Infinite M200 microplate reader spectrophotometer (Tecan, Graz, Austria). The TAC was expressed as the Trolox equivalent antioxidant capacity concentration (mM).

#### 2.4.5. 8-Isoprostane (8-Iso-PGF2α)

Lipid peroxidation was assessed in urine samples using an immunoassay of 8-isoprostane concentration (Cayman Chemical, Ann Arbor, MI, USA, Item No. 516351) [29,30]. Briefly, 50 μL urine samples were added to a 96-well plate pre-coated with mouse monoclonal antibody, followed by 50 μL of 8-iso PGF2α-tracer antiserum, and incubated. After washing, 200 μL of Ellman’s reagent was added. The samples and standard were read in duplicate at a wavelength of 412 nm. The results were normalized using urine creatinine values. The coefficient of variation (CV) indicated by the manufacturer was as follows: inter-assay CV 11.5%; intra-assay CV 8.9%.

#### 2.4.6. NO Metabolites

The NO derivatives nitrate and nitrite (NO_2_ + NO_3_ = NOx) were measured in the urine samples by a colorimetric method based on the Griess reaction, using a commercial kit (Cayman Chemical, Ann Arbor, MI, USA; Item No. 780001), [31,32]. The samples were read at 545 nm, and the concentration was assessed by a standard curve. The coefficients of variation (CV) indicated by the manufacturer were as follows: inter-assay CV: 3.4%; intra-assay CV: 2.7%.

#### 2.4.7. Co Q10 Coenzyme

The CoQ10 plasma levels were quantified using the Human Coenzyme Q10 Elisa Kit (EKC33185, Biomatik, ON, Canada), following the manufacturer’s instructions. Briefly, the assay employs the competitive inhibition enzyme immunoassay technique; the microtiter plate provided in this kit has been pre-coated with CoQ10. Standards or plasma samples are added to the appropriate microtiter plate wells with a Horseradish Peroxidase (HRP) conjugated antibody preparation specific to CoQ10. The competitive inhibition reaction is launched between with pre-coated CoQ10 and CoQ10 in samples. The samples were read at 450 nm, and the concentration was assessed using a standard curve.

#### 2.4.8. Thiols Measurement

Total (tot) and reduced (red) aminothiols (Cys = cysteine, and GSH = glutathione) were measured in erythrocytes (RBCs) according to previously validated methods [33,34,35]. Thiol separation was performed at room temperature via isocratic high-pressure liquid chromatography (HPLC) analysis on a Discovery C-18 column (250 × 4.6 mm I.D, Supelco, Sigma-Aldrich, St. Louis, MO, USA), eluted with a solution of 0.1 M acetate buffer, pH 4.0: methanol, 81:19 (*v*/*v*), at a flow rate of 1 mL/min. The fluorescence intensities were measured with an excitation wavelength at 390 nm and an emission wavelength at 510 nm, using a fluorescence spectrophotometer (Jasco, Tokyo, Japan). A standard calibration curve was used.

#### 2.4.9. Creatinine, Neopterin, and Uric Acid

The urinary creatinine, neopterin, and uric acid concentrations were measured in urine by isocratic high-pressure liquid chromatography (HPLC) [36,37,38]. Briefly, the urine samples were thawed and centrifuged at 13,000 rpm for 5 min at 4 °C; the supernatant was then adequately diluted with chromatographic mobile phase (15 mM of K_2_HPO_4_, pH 3.0).

Neopterin, creatinine and uric acid were measured using a Varian pump (240, auto sampler ProStar 410; Markham, ON, Canada) coupled to a fluorometric detector (JASCO FP-1520, λex = 355 nm and at λem = 450 nm; Burladingen, Germany) for neopterin and to a UV-VIS detector (Shimadzu SPD 10-AV, λ = 240 nm; Duisburg, Germany) for the creatinine and uric acid determinations. The calibration curves were linear over the range of 0.125–1 μmol/L, 0.625–20 mmol/L, and 1.25–10 mmol/L for the neopterin, uric acid, and creatinine levels, respectively. The inter-assay and intra-assay coefficients of variation were <5%.

### 2.5. Statistic Analysis

The Kolmogorov–Smirnov test was implemented to assess whether each variable followed a normal distribution, and descriptive statistics were calculated. A two-way ANOVA with the factors “type product” (Active vs. Placebo) and “time” (1° day vs. 7° day) was applied. Then, the one-way ANOVA was applied to compare the trend value between the two groups. Tukey’s honest tests were used for post hoc analysis. Probability levels of <0.05 were considered significant. Throughout the text, values are expressed as mean ± standard deviation of the mean (SD). The correlations between the investigated variables were assessed using Spearman correlation coefficients. Change ∆% estimation (([post value-pre value]/pre value) × 100) was also reported in the text. Statistical analysis was performed using the GraphPad Prism package for Mac (GraphPad Prism 9.5.1, GraphPad Software Inc., San Diego, CA, USA). ROS production was considered as the primary outcome (no other parameters were taken into account), and prospective calculations of power to determine the sample size were made using G power software (GPower 3.1) [39]. At 80% power, the sample size—calculated in preliminary studies [40,41,42]—was set at eleven/thirteen subjects.

## 3. Results

No dropouts were recorded. In the investigated subjects, no significant alterations in MCV, AST, ALT, γ GT, and total and fractionated bilirubin were detected (Table 1).

### 3.1. Effects of Product Intake on Ethanol Concentration during the Acute Phase

**Figure 2:**
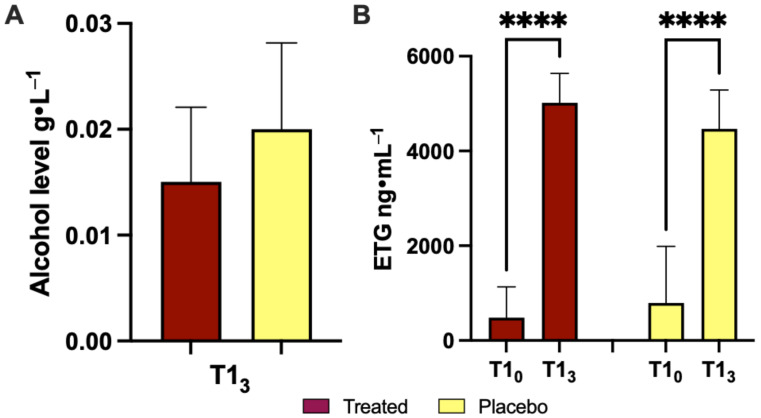
(**A**) Alcohol level at 120 min after treatment (Si.Pi.Mi. in red) with respect to placebo intake (T1_3_; 1st day; in yellow); (**B**) ETG level 120 min after treatment or placebo intake (T1_3_; 1st day). Statistically significant differences are displayed as ****, *p* < 0.001.

Figure 2A and Table 2 show the alcohol concentration (g/L) on the 1st day in subjects who consumed the placebo or Si.Pi.Mi. after wine. Data were recorded after 120 min, with an increase in the placebo (+33%) vs. treated patients. Figure 2B and Table 2 report the ETG value (ng/mL), an endogenous metabolic product of ethanol formed by its conjugation with glucuronic acid, measured 120 min after intake on day 1. A statistically significant difference (*p* < 0.0001) in the ETG value was observed in both groups before and after treatment or placebo intake, while no significant difference between the post-ETG level in the two groups was observed.

### 3.2. Effects of Product Intake on Oxidative Stress in Short and Long Phase

**Figure 3:**
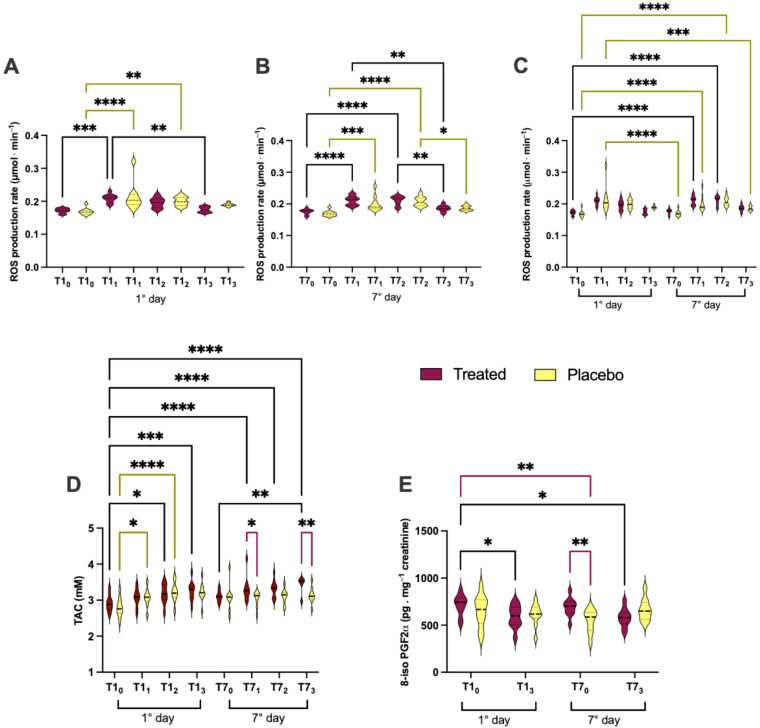
(**A**–**C**) ROS production, (**D**) TAC concentration assessed in plasma and (**E**) 8-iso-PGF2α biomarker of lipid peroxidation. Levels detected in urine, after treatment (in red) or placebo (in yellow) intake on the 1st and 7th day. Statistically significant intra (treaded: black lines; placebo: yellow line) and inter-group (purple lines) differences are displayed as follows: * *p* < 0.05; ** *p* < 0.01; *** *p* < 0.001; **** *p* < 0.0001.

The ROS production rate, TAC in plasma and oxidative stress biomarker concentrations of lipid peroxidation (8-iso-PGF2α) in urine, assessed in subjects after treatment or placebo intake on the 1st and 7th day, are displayed in Figure 3. The statistically significant differences, intra and inter-group, calculated from the data collected between different groups, are herein reported. In particular (Figure 3A,B), a significantly different ROS production level (µmol·min^−1^) between treatment and placebo groups can be observed. In detail, significant ROS differences (µmol·min^−1^) were calculated as follows:

(i) in treated group
  1st day: T1_0_ vs. T1_1_ (0.171 ± 0.007 vs. 0.208 ± 0.012); T1_1_ vs. T1_3_ (0.208 ± 0.012 vs. 0.174 ± 0.008);
  7th day: T7_0_ vs. T7_1_ (0.175 ± 0.008 vs. 0.210 ± 0.014); T7_0_ vs. T7_2_ (0.175 ± 0.008 vs. 0.210 ± 0.015); T7_1_ vs. T7_3_ (0.210 ± 0.014vs 1.186 ± 0.009); T7_2_ vs. T7_3_ (0.210 ± 0.015vs 1.186 ± 0.009);
  1st vs. 7th day: T1_0_ vs. T7_1_ (0.171 ± 0.007 vs. 0.210 ± 0.014vs); and T1_0_ vs. T7_2_ (0.171 ± 0.007 vs. 0.210 ± 0.015);
(ii) in placebo group
  1st day: T1_0_ vs. T1_1_ (0.169 ± 0.009 vs. 0.212 ± 0.040); T1_1_ vs. T1_2_ (0.212 ± 0.040 vs. 0.199 ± 0.013); T7_0_ vs. T7_1_ (0.169 ± 0.008 vs. 0.200 ± 0.023);
  7th day: T7_0_ vs. T7_2_ (0.169 ± 0.008 vs. 0.207 ± 0.015); T7_0_ vs. T7_3_ (0.169 ± 0.008 vs. 0.186 ± 0.007); T7_2_ vs. T7_3_ (0.207 ± 0.015 vs. 0.186 ± 0.007); T1_0_ vs. T7_1_ (0.169 ± 0.009 vs. 0.200 ± 0.023);
  1st vs. 7th day: T1_0_ vs. T7_2_ (0.169 ± 0.009 vs. 0.207 ± 0.015); T1_1_ vs. T7_0_ (0.212 ± 0.040 vs. 0.169 ± 0.008); and T7_1_ vs. T7_3_ (0.200 ± 0.023vs 0.186 ± 0.007);
(iii) inter-group (treated vs. placebo): no significant differences.

In TAC (mM; Figure 3D), there were significant differences between the treatment and placebo groups, as follows:

(i) in the treated group
  1st day: T1_0_ vs. T1_2_ (2.911 ± 0.227 vs. 3.176 ± 0.269); T1_0_ vs. T1_3_ (2.911 ± 0.227 vs. 3.255 ± 0.265);
  7th day: T7_0_ vs. T7_3_ (3.109 ± 0.153 vs. 3.420 ± 0.241);
  1st vs. 7th day: T1_0_ vs. T7_1_ (2.911 ± 0.227 vs. 3.304 ± 0.333); T1_0_ vs. T7_2_ (2.911 ± 0.227 vs. 3.315 ± 0.231); T1_0_ vs. T7_3_ (2.911 ± 0.227 vs. 3.420 ± 0.241);
(ii) in placebo group
  1st day: T1_0_ vs. T1_1_ (2.799 ± 0.258 vs. 3.068 ± 0.241); and T1_0_ vs. T1_2_ (2.799 ± 0.258 vs. 3.224 ± 0.269);
(iii) inter-group (treated vs. placebo): at T7_1_ (3.109 ± 0.153 vs. 3.083 ± 0.353); and T7_3_ (3.420 ± 0.241 vs. 3.130 ± 0.209).

Concerning 8-iso-PGF2α (pg⋅mg^−1^creatinine; Figure 3E), there were significant differences between the treated and placebo groups, as follows:

(i) in the treated group: T1_0_ vs. T1_3_ (793.3 ± 120.6 vs. 617.5± 131.0); and T1_0_ vs. T7_3_ (793.3 ± 120.6 vs. 579.9 ± 100.4);
(ii) in placebo group no significant differences;
(iii) inter-group (treated vs. placebo): at T7_0_ (793.3 ± 120.6 vs. 651.7 ± 165.6), and T1_0_ vs. T7_0_ (793.3 ± 120.6 vs. 545.7 ± 122.0).

**Figure 4:**
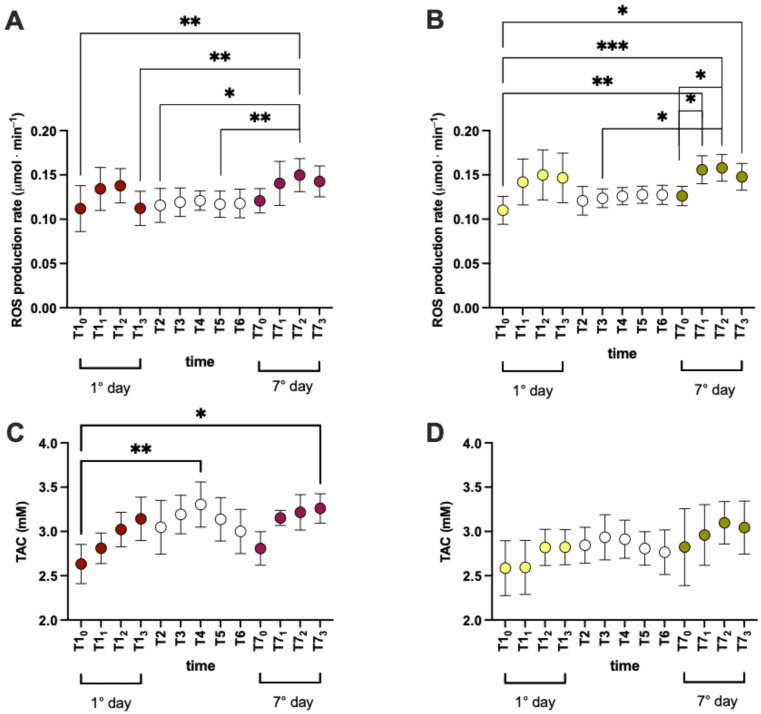
Time course (mean ± SD) of (**A**,**B**) Reactive Oxygen Species (ROS) production and (**C**,**D**) Total Antioxidant Capacity (TAC) detected on saliva in ten subjects (five treated-red/white + five placebo-yellow/white). Significant intra-group differences: * *p* < 0.05; ** *p* < 0.01; *** *p* < 0.001.

In ten subjects (*n* = 5 treated and *n* = 5 placebo), the ROS production and TAC were also measured in saliva samples during the experimental week to study the evolution. Changes in the ROS levels were registered during the experimental session, and increases/decreases were observed in the short and long phase. In particular, during the time course recorded on the 1st day and 7th day, increases in ROS (Figure 4A,B) and the TAC (Figure 4C,D) were recorded.

No significant differences were found in NO metabolites in both groups.

### 3.3. CoQ10 Coenzyme

**Figure 5:**
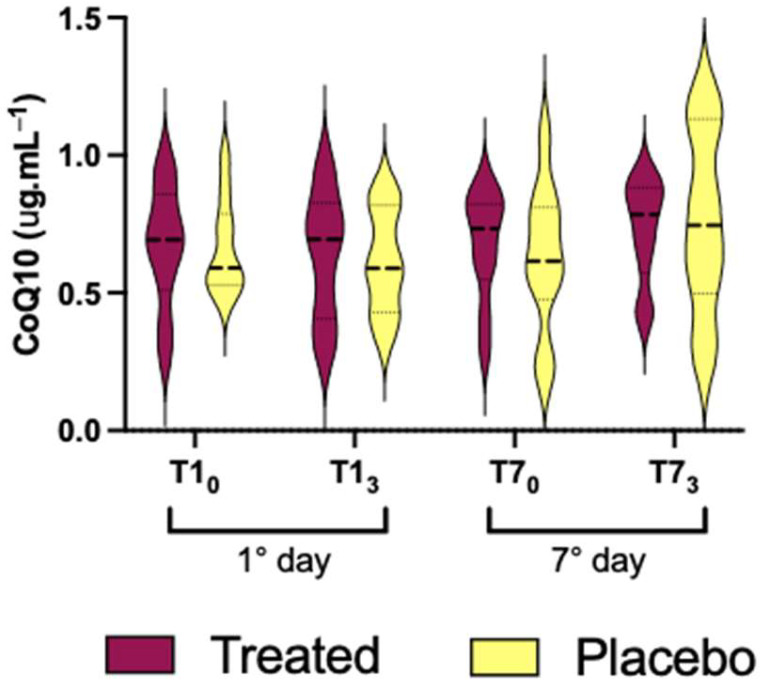
Co Q10 plasma value after treatment (Si.Pi.Mi. in red) or placebo (yellow) intake on the 1st and 7th day.

Not significant differences were found in the Co Q10 plasma levels between the intra/inter groups (treated vs. placebo; Figure 5).

### 3.4. Redox Status

**Figure 6:**
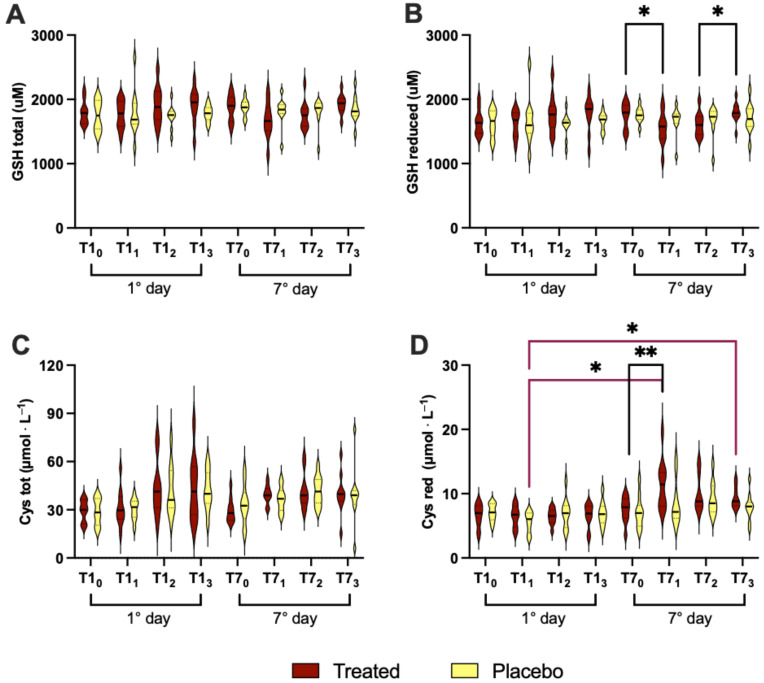
Total and reduced GSH (**A**,**B**) and cysteine (**C**,**D**) values after treatment (Si.Pi.Mi. in red) or placebo (yellow) intake on the 1st and 7th day. Statistically significant intra (treated: black line) and inter-group (purple lines) differences are displayed as * *p* < 0.05; ** *p* < 0.01.

Amino thiols analysis was performed in RBC using HPLC techniques to evaluate the redox status of the subjects. We did not find any statistically significant differences in the total GSH and cysteine between the treatment and placebo groups (Figure 6A,C). Furthermore, reduced GSH showed a significant difference (*p* < 0.05) on the 7th day in the treatment group (*p* < 0.05; T7_0_ vs. T7_1_ (−13%) and T7_2_ vs. T7_3_ (+12%); Figure 6B); meanwhile, reduced cysteine exhibited a statistically significant difference on the 7th day in the treated group (*p* < 0.01; T7_0_ vs. T7_1_: +57%), and in the intragroup for the T1_1_ placebo with respect to the T7_1_ and T7_3_ treatment (−10% and +6%, respectively) (Figure 6D).

### 3.5. Neopterin and Uric Acid Concentration

**Figure 7:**
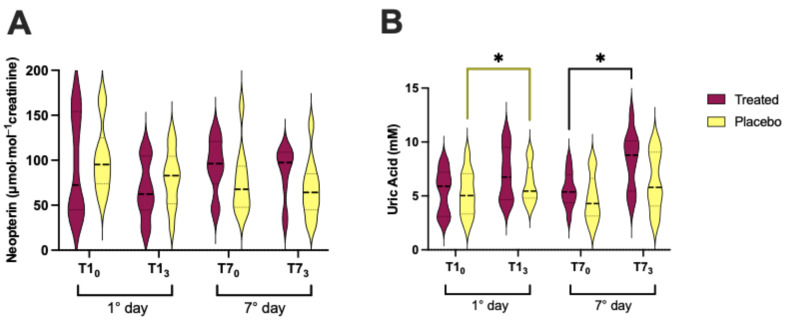
(**A**) Neopterin and (**B**) uric acid levels after treatment (Si.Pi.Mi. in red) intake vs. placebo (yellow). Statistically significant intra differences (treated: black line; placebo: yellow lines) are displayed as * *p* < 0.05.

Urine samples were investigated to assess the neopterin and uric acid (Figure 7) concentrations.

The neopterin concentration did not show significant differences in both groups (Figure 7A). Concerning uric acid, the data showed an increase on the 1st day in the placebo (*p* < 0.05; +17%; Figure 7B) and on the 7th day in the treated subjects (*p* < 0.05; +48%; Figure 7B).

### 3.6. Data Correlation

**Figure 8:**
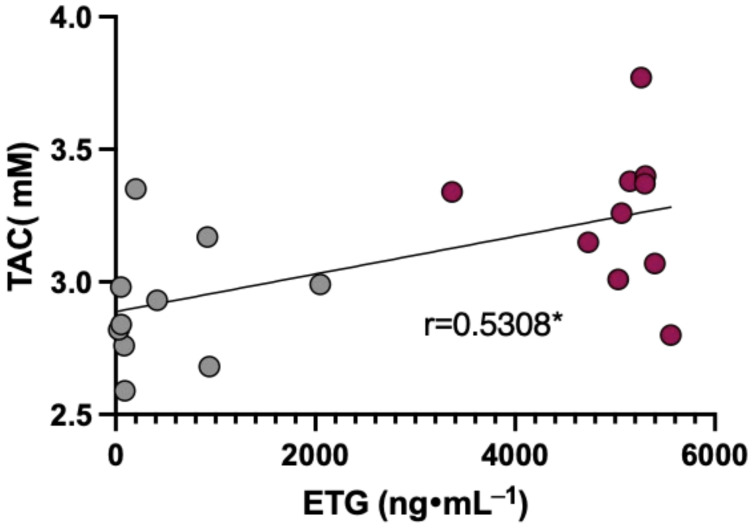
Plots of the TAC concentration (mM) measured in plasma (in grey) versus ETG (ng·mL^−1^) measured in urine (in red). The linear regression fit (solid line) shows the correlation coefficient (r). A significant linear relationship (* < 0.05) was estimated.

Lastly, a possible correlation between biomarkers, ETG and alcohol data were investigated on the 1st day: T1_0_ (grey) vs. T1_3_ (purple) after Si.Pi.Mi. intake. The plot reported in Figure 8 shows a significant linear relationship (r = 0.5308) between the plasma TAC concentration and urine ETG level.

## 4. Discussion

This study aimed to investigate the efficacy of Si.Pi.Mi in reducing circulating ethanol and oxidative stress biomarkers.

Figure 2A shows a greater decrease (−33%) in the blood ethanol concentration in subjects who took Si.Pi.Mi with respect to the placebo group, which may be the consequence of the effects of one or more components. Silymarin, a mixture of flavolignans, may act as a hepatoprotector against EtOH-induced liver damage, retarding the development of alcohol-induced hepatic fibrosis in an animal model [43]. However, in animals, cirrhosis is different from the alcoholic liver fibrosis produced in humans and human trials. Some authors observed that silymarin supplementation was not influenced by alcohol intake or the severity of liver dysfunction, and did not have any significant effect on the time course of the disease [44,45]. On the other hand, PQQ seems to stimulate ADH and ALDH activity, improving the hepatic detoxification process, and alleviate the alcohol-induced damage of liver cells [19]. The ETG increased in both groups (Figure 2B). In the placebo group, this could be ascribed to incidental exposure to ethanol: the sources of possible exposure in the environment include alcohol in mouthwash, foods and medications, and even the inhalation of alcohol from topical use [46].

Excessive ethanol consumption can promote and exacerbate oxidative stress in the liver, reduce the endogenous antioxidant defense capacity by scavenging ROS, and accumulate oxidation products in the body, leading to liver damage [47]. Moreover, the concentration of oxidants can be increased through several pathways, including mitochondrial oxidative phosphorylation, the cellular activation of NADPH oxidase/xanthine oxidase (NOX/XOX), nitric oxide synthase (NOS), and a copper/iron-catalyzed Fenton–Weiss-Haber reaction of H_2_O_2_ [48].

As reported in Figure 3 and Figure 4, the plasma and salivary ROS production kinetics exhibited the same trend, but we observed a greater increase in the TAC level in the Si.Pi.Mi. salivary kinetics versus the placebo kinetics (Figure 4C,D) and between the first and the last experimental day (Figure 4C). Saliva is the first line of defense against ROS and RNS [49], associated with an antioxidant mechanism that involves glutathione, ascorbic, uric acid, melatonin [50] and the enzymes CAT, SOD and GPx [51]. As observed in the plasma samples, Si.Pi.Mi. may activate some of these salivary antioxidants to avoid macromolecule damage and the onset of oral inflammatory diseases such as periodontitis [52]. Silymarin seems to improve mitochondrial metabolic processes and the electron transport chain, increasing SOD activity and reducing monoamine oxidase (MAO) expression, leading to a reduction in ROS levels [53,54].

It was reported that silymarin could reduce the endogenous antioxidant depletion induced by acute EtOH administration, so one of the major effects of silymarin in acute EtOH-induced liver injury might result from its direct free radical scavenging capacity and ability to prevent increases in alanine transferase (ALT) and tumor necrosis factor-α (TNF-α) [17]. Indeed, silymarin is able to reduce the reactive substance of thiobarbituric acid (TBARS) and the lipid peroxidation index, and improve SOD, CAT, glutathione reductase (GR) and GPx, which are related to EtOH exposure [55]. PQQ may reduce MDA, another well-known lipid peroxidation marker, and enhance the activities of antioxidant enzymes including CAT and SOD, thus increasing antioxidant defenses [19,56]. Furthermore, myricetin can reduce TGF-β1 generation, decreasing oxidative stress and preventing alcohol-induced lipid accumulation [57].

Concerning 8-isoprostane (Figure 3E), the data showed a significant decrease after Si.Pi.Mi. intake, while uric acid increased at day 7 (Figure 7B) according to the significant increase in the TAC (Figure 3D), corroborating the supposed antioxidant properties of Si.Pi.Mi. Indeed, some authors observed a decrease in 8-isoprostane as a consequence of silymarin consumption [58].

The effect of a higher TAC is also pointed out by the correlation between the TAC and ETG on the 1st day, 3 h after Si.Pi.Mi. administration (Figure 8). This suggests that the antioxidant capacity is able to avoid alcohol-metabolism-related ROS production, hepatocyte injury, and cell apoptosis [59,60].

CoQ10 (Figure 5) did not exhibit any statistically significant difference in both groups, probably due to the sample’s SD and size. In our case, it is possible that ethanol intake was not enough to deplete mitochondrial coenzyme-Q10 [61].

We did not observe any change in total GSH (Figure 6A) between the Si.Pi.Mi. and placebo groups, observing a constant trend for the duration of the experiment. On the other hand, reduced GSH decreased between the first and seventh day of the experiment (Figure 6B). Reduced GSH is the main antioxidant found in cells, playing a key role in the protection against several toxic agents, including ethanol [62]. Some authors found that both acute and chronic exposure to ethanol leads to a time and dose-dependent decrease in the hepatic GSH level [63]. Furthermore, a decrease in GSH can be associated with ethanol toxicity, which may reflect the consumption of GSH by the overgeneration of ROS [64,65]. 

On the other hand, the increase in the cysteine level in the Si.Pi.Mi. group might be ascribed to the decrease in GSH (Figure 6). Due to its ability to undergo redox reactions, cysteine shows antioxidant properties, being necessary for glutathione biosynthesis [66], a component of the body’s antioxidant defenses.

## 5. Limitation

This study suffers from some limitations. These are mainly due to the limited sample size and the high inter-individual variability of the examined subjects; many significances could not show up, and a correlation between the alcohol level and other biomarkers was not allowed. By including a broader and/or more diverse population, the study would be better designed to reflect the effects of alcohol consumption across different demographics. Moreover, by adjusting the dosage by increasing the amount of alcohol administered—specifically, the number of glasses of wine per day—we could provide a clearer understanding of the relationship between alcohol intake and its effects. This approach would allow us to explore potential dose-dependent outcomes, adding new perspectives on the study’s findings.

Future studies should consider subjects who are habitual alcohol consumers, focusing on moderate vs. high wine consumption, and occasional vs. chronic consumption. Additionally, subjects with different BMIs should be considered, and the sample should be expanded to include other ethnic groups. Finally, further intervention studies should be performed to explore the effect of Si.Pi.Mi. on the intake of alcoholic beverages other than red wine.

## 6. Conclusions

Si.Pi.Mi. reduced circulating ethanol after 120 min, probably due to its antioxidant components (silymarin, PQQ, and myricetin). The data reported in this study agree with other data available in the literature reporting ethanol [67], smoke [68] and pollution [69] as main exogenous ROS sources. The only effect of ethanol was taken into account in the present study: there were no smokers among the subjects, nor were environments with different pollution types investigated.

The raised blood ethanol led to an increase in ROS production, while Si.Pi.Mi. intake promoted the activation of endogenous antioxidants, as evidenced by the increase in TAC levels and the reduction in 8-isoprostane. The CoQ10 levels remained unchanged but the decrease in reduced GSH and corresponding increase in cysteine might suggest that Si.Pi.Mi plays a role in modulating antioxidant pathways. These findings indicate that Si.Pi.Mi. might support hepatic detoxification and protect against alcohol-induced oxidative damage.

## Figures and Tables

**Table 1:** Laboratory parameters: MCV = Mean Cell Volume; AST = Aspartate Aminotransferase; ALT = Alanine Transaminase; γ-GT = gamma-glutamyl transferase; TBL = Total Bilirubin; DBIL = Direct Bilirubin; UBIL = Indirect Bilirubin. Data are expressed as mean values (±SD).

Parameter	T0		T7	
	Treated	Placebo	Treated	Placebo
**MCV (fL)** range * (80–100)	90.4 ± 2.7	90.2 ± 3.0	90.4 ± 2.1	90.0 ± 3.2
**AST (U/L)** **Range (17–59)**	32.8 ± 10.1	23.8 ± 9.8	32.0 ± 17.0	20.8 ± 5.1
**ALT (U/L)** **Range (7–55)**	21.8 ± 10.8	19.2 ± 10.0	28.2 ± 34.7	15.4 ± 8.6
**γ-GT (U/L)** **range (5–40)**	15.2 ± 4.3	10.6 ± 7.7	16.4 ± 3.8	13.8 ± 4.7
**TBL (mg/dL)** **Range (0.1–1.2)**	0.67 ± 0.09	0.56 ± 0.07	1.03 ± 0.35	0.47 ± 0.15
**DBIL (mg/dL)** **Range (0.1–0.3)**	0.25 ± 0.07	0.24 ± 0.10	0.26 ± 0.14	0.25 ± 0.11
**UBIL (mg/dL)** **Range (0.2–0.8)**	0.39 ± 0.40	0.36 ± 0.22	0.41 ± 0.25	0.37 ± 0.11

* This refers to the range of normal values.

**Table 2:** Alcohol concentration (g/L) and ETG level (ng/mL) in treated and placebo groups at T1_0_ and T1_3_. Data are expressed as mean values (±SD).

Parameter	T1_0_		T1_3_	
	Treated	Placebo	Treated	Placebo
**Alcohol level (g/L)**	-	-	0.015 ± 0.007	0.020 ± 0.008
**ETG (ng/mL)**	483.1 ± 650.9	790.4 ± 1197	5017 ± 622.1	4466 ± 823.6

## Data Availability

Data are contained within the article.

## References

[1] Fuster D., Samet J.H. (2018). Alcohol Use in Patients with Chronic Liver Disease. N. Engl. J. Med..

[2] Shen N.T., Salajegheh A., Brown R.S. (2019). A Call to Standardize Definitions, Data Collection, and Outcome Assessment to Improve Care in Alcohol-Related Liver Disease. Hepatology.

[3] Plapp B.V. (1975). Rate-limiting steps in ethanol metabolism and approaches to changing these rates biochemically. Adv. Exp. Med. Biol..

[4] Loguercio C., Federico A. (2003). Oxidative stress in viral and alcoholic hepatitis. Free Radic. Biol. Med..

[5] Albano E. (2008). Oxidative mechanisms in the pathogenesis of alcoholic liver disease. Mol. Aspects Med..

[6] Munzel T., Gori T., Bruno R.M., Taddei S. (2010). Is oxidative stress a therapeutic target in cardiovascular disease?. Eur. Heart J..

[7] Pacher P., Beckman J.S., Liaudet L. (2007). Nitric oxide and peroxynitrite in health and disease. Physiol. Rev..

[8] Arosio P., Levi S. (2002). Ferritin, iron homeostasis, and oxidative damage. Free Radic. Biol. Med..

[9] Reed D.J. (2004). Mitochondrial glutathione and chemically induced stress including ethanol. Drug Metab. Rev..

[10] Tug T., Karatas F., Terzi S.M. (2004). Antioxidant vitamins (A, C and E) and malondialdehyde levels in acute exacerbation and stable periods of patients with chronic obstructive pulmonary disease. Clin. Investig. Med..

[11] Kono H., Bradford B.U., Yin M., Sulik K.K., Koop D.R., Peters J.M., Gonzalez F.J., McDonald T., Dikalova A., Kadiiska M.B. (1999). CYP2E1 is not involved in early alcohol-induced liver injury. Am. J. Physiol..

[12] Dawson D.A. (2003). Methodological issues in measuring alcohol use. Alcohol Res. Health.

[13] Jones A.W., Andersson L. (1996). Variability of the blood/breath alcohol ratio in drinking drivers. J. Forensic Sci..

[14] Verster J.C., Penning R. (2010). Treatment and prevention of alcohol hangover. Curr. Drug Abus. Rev..

[15] Liang X.T., Wang Y.Y., Hu X.Y., Wang S.B. (2021). The Protective Effects of Water Extracts of Compound Turmeric Recipe on Acute Alcoholism: An Experimental Research Using a Mouse Model. Evid.-Based Complement. Altern. Med..

[16] Chambers C.S., Holeckova V., Petraskova L., Biedermann D., Valentova K., Buchta M., Kren V. (2017). The silymarin composition... and why does it matter???. Food Res. Int..

[17] Song Z., Deaciuc I., Song M., Lee D.Y., Liu Y., Ji X., McClain C. (2006). Silymarin protects against acute ethanol-induced hepatotoxicity in mice. Alcohol Clin. Exp. Res..

[18] Guo C., Xue G., Pan B., Zhao M., Chen S., Gao J., Chen T., Qiu L. (2019). Myricetin Ameliorates Ethanol-Induced Lipid Accumulation in Liver Cells by Reducing Fatty Acid Biosynthesis. Mol. Nutr. Food Res..

[19] Jiang X., Zhou Y., Zhang Y., Tian D., Jiang S., Tang Y. (2020). Hepatoprotective effect of pyrroloquinoline quinone against alcoholic liver injury through activating Nrf2-mediated antioxidant and inhibiting TLR4-mediated inflammation responses. Process Biochem..

[20] Sarafian D., Maufrais C., Montani J.P. (2018). Early and Late Cardiovascular and Metabolic Responses to Mixed Wine: Effect of Drink Temperature. Front. Physiol..

[21] Baraona E., Abittan C.S., Dohmen K., Moretti M., Pozzato G., Chayes Z.W., Schaefer C., Lieber C.S. (2001). Gender differences in pharmacokinetics of alcohol. Alcohol. Clin. Exp. Res..

[22] World Medical A. (2013). World Medical Association Declaration of Helsinki: Ethical principles for medical research involving human subjects. JAMA.

[23] Kerr W.C., Greenfield T.K., Tujague J., Brown S.E. (2005). A drink is a drink? Variation in the amount of alcohol contained in beer, wine and spirits drinks in a US methodological sample. Alcohol. Clin. Exp. Res..

[24] Savini F., Tartaglia A., Coccia L., Palestini D., D’Ovidio C., de Grazia U., Merone G.M., Bassotti E., Locatelli M. (2020). Ethanol Determination in Post-Mortem Samples: Correlation between Blood and Vitreous Humor Concentration. Molecules.

[25] Mrakic-Sposta S., Gussoni M., Montorsi M., Porcelli S., Vezzoli A. (2012). Assessment of a standardized ROS production profile in humans by electron paramagnetic resonance. Oxidative Med. Cell. Longev..

[26] Mrakic-Sposta S., Gussoni M., Montorsi M., Porcelli S., Vezzoli A. (2014). A quantitative method to monitor reactive oxygen species production by electron paramagnetic resonance in physiological and pathological conditions. Oxidative Med. Cell. Longev..

[27] Mrakic-Sposta S., Gussoni M., Moretti S., Pratali L., Giardini G., Tacchini P., Dellanoce C., Tonacci A., Mastorci F., Borghini A. (2015). Effects of Mountain Ultra-Marathon Running on ROS Production and Oxidative Damage by Micro-Invasive Analytic Techniques. PLoS ONE.

[28] Mrakic-Sposta S., Vezzoli A., Garetto G., Paganini M., Camporesi E., Giacon T.A., Dellanoce C., Agrimi J., Bosco G. (2023). Hyperbaric Oxygen Therapy Counters Oxidative Stress/Inflammation-Driven Symptoms in Long COVID-19 Patients: Preliminary Outcomes. Metabolites.

[29] Strapazzon G., Malacrida S., Vezzoli A., Dal Cappello T., Falla M., Lochner P., Moretti S., Procter E., Brugger H., Mrakic-Sposta S. (2016). Oxidative stress response to acute hypobaric hypoxia and its association with indirect measurement of increased intracranial pressure: A field study. Sci. Rep..

[30] Mrakic-Sposta S., Gussoni M., Dellanoce C., Marzorati M., Montorsi M., Rasica L., Pratali L., D’Angelo G., Martinelli M., Bastiani L. (2021). Effects of acute and sub-acute hypobaric hypoxia on oxidative stress: A field study in the Alps. Eur. J. Appl. Physiol..

[31] Green L.C., Wagner D.A., Glogowski J., Skipper P.L., Wishnok J.S., Tannenbaum S.R. (1982). Analysis of nitrate, nitrite, and [15N] nitrate in biological fluids. Anal. Biochem..

[32] Brizzolari A., Bosco G., Vezzoli A., Dellanoce C., Barassi A., Paganini M., Cialoni D., Mrakic-Sposta S. (2023). Seasonal Oxy-Inflammation and Hydration Status in Non-Elite Freeskiing Racer: A Pilot Study by Non-Invasive Analytic Method. Int. J. Environ. Res. Public Health.

[33] Dellanoce C., Cozzi L., Zuddas S., Pratali L., Accinni R. (2014). Determination of different forms of aminothiols in red blood cells without washing erythrocytes. Biomed. Chromatogr..

[34] Vezzoli A., Dellanoce C., Mrakic-Sposta S., Montorsi M., Moretti S., Tonini A., Pratali L., Accinni R. (2016). Oxidative Stress Assessment in Response to Ultraendurance Exercise: Thiols Redox Status and ROS Production according to Duration of a Competitive Race. Oxidative Med. Cell. Longev..

[35] Biswas P., Dellanoce C., Vezzoli A., Mrakic-Sposta S., Malnati M., Beretta A., Accinni R. (2020). Antioxidant Activity with Increased Endogenous Levels of Vitamin C, E and A Following Dietary Supplementation with a Combination of Glutathione and Resveratrol Precursors. Nutrients.

[36] Mrakic-Sposta S., Vezzoli A., Rizzato A., Della Noce C., Malacrida S., Montorsi M., Paganini M., Cancellara P., Bosco G. (2019). Oxidative stress assessment in breath-hold diving. Eur. J. Appl. Physiol..

[37] Glantzounis G.K., Tsimoyiannis E.C., Kappas A.M., Galaris D.A. (2005). Uric acid and oxidative stress. Curr. Pharm. Des..

[38] Demartini B., Nistico V., Benayoun C., Cigognini A.C., Ferrucci R., Vezzoli A., Dellanoce C., Gambini O., Priori A., Mrakic-Sposta S. (2023). Glutamatergic dysfunction, neuroplasticity, and redox status in the peripheral blood of patients with motor conversion disorders (functional movement disorders): A first step towards potential biomarkers discovery. Transl. Psychiatry.

[39] Faul F., Erdfelder E., Lang A.G., Buchner A. (2007). G*Power 3: A flexible statistical power analysis program for the social, behavioral, and biomedical sciences. Behav. Res. Methods.

[40] Mrakic-Sposta S., Vezzoli A., Maderna L., Gregorini F., Montorsi M., Moretti S., Greco F., Cova E., Gussoni M. (2018). R(+)-Thioctic Acid Effects on Oxidative Stress and Peripheral Neuropathy in Type II Diabetic Patients: Preliminary Results by Electron Paramagnetic Resonance and Electroneurography. Oxidative Med. Cell. Longev..

[41] Bosco G., Paganini M., Giacon T.A., Oppio A., Vezzoli A., Dellanoce C., Moro T., Paoli A., Zanotti F., Zavan B. (2021). Oxidative Stress and Inflammation, MicroRNA, and Hemoglobin Variations after Administration of Oxygen at Different Pressures and Concentrations: A Randomized Trial. Int. J Environ. Res. Public Health.

[42] Vezzoli A., Mrakic-Sposta S., Dellanoce C., Montorsi M., Vietti D., Ferrero M.E. (2023). Chelation Therapy Associated with Antioxidant Supplementation Can Decrease Oxidative Stress and Inflammation in Multiple Sclerosis: Preliminary Results. Antioxidants.

[43] Lieber C.S., Leo M.A., Cao Q., Ren C., DeCarli L.M. (2003). Silymarin retards the progression of alcohol-induced hepatic fibrosis in baboons. J. Clin. Gastroenterol..

[44] Pares A., Planas R., Torres M., Caballeria J., Viver J.M., Acero D., Panes J., Rigau J., Santos J., Rodes J. (1998). Effects of silymarin in alcoholic patients with cirrhosis of the liver: Results of a controlled, double-blind, randomized and multicenter trial. J. Hepatol..

[45] Lucena M.I., Andrade R.J., de la Cruz J.P., Rodriguez-Mendizabal M., Blanco E., Sanchez de la Cuesta F. (2002). Effects of silymarin MZ-80 on oxidative stress in patients with alcoholic cirrhosis. Results of a randomized, double-blind, placebo-controlled clinical study. Int. J. Clin. Pharmacol. Ther..

[46] Rosano T.G., Lin J. (2008). Ethyl glucuronide excretion in humans following oral administration of and dermal exposure to ethanol. J. Anal. Toxicol..

[47] Xia T., Zhang J., Yao J., Zhang B., Duan W., Zhao C., Du P., Song J., Zheng Y., Wang M. (2018). Shanxi Aged Vinegar Protects against Alcohol-Induced Liver Injury via Activating Nrf2-Mediated Antioxidant and Inhibiting TLR4-Induced Inflammatory Response. Nutrients.

[48] Haorah J., Ramirez S.H., Floreani N., Gorantla S., Morsey B., Persidsky Y. (2008). Mechanism of alcohol-induced oxidative stress and neuronal injury. Free Radic. Biol. Med..

[49] Amerongen A.V., Veerman E.C. (2002). Saliva-the defender of the oral cavity. Oral Dis..

[50] Balaji T.M., Vasanthi H.R., Rao S.R. (2015). Gingival, plasma and salivary levels of melatonin in periodontally healthy individuals and chronic periodontitis patients: A pilot study. J. Clin. Diagn. Res..

[51] Battino M., Ferreiro M.S., Gallardo I., Newman H.N., Bullon P. (2002). The antioxidant capacity of saliva. J. Clin. Periodontol..

[52] Sczepanik F.S.C., Grossi M.L., Casati M., Goldberg M., Glogauer M., Fine N., Tenenbaum H.C. (2020). Periodontitis is an inflammatory disease of oxidative stress: We should treat it that way. Periodontology.

[53] Detaille D., Sanchez C., Sanz N., Lopez-Novoa J.M., Leverve X., El-Mir M.Y. (2008). Interrelation between the inhibition of glycolytic flux by silibinin and the lowering of mitochondrial ROS production in perifused rat hepatocytes. Life Sci..

[54] Grattagliano I., Diogo C.V., Mastrodonato M., de Bari O., Persichella M., Wang D.Q., Liquori A., Ferri D., Carratu M.R., Oliveira P.J. (2013). A silybin-phospholipids complex counteracts rat fatty liver degeneration and mitochondrial oxidative changes. World J. Gastroenterol..

[55] Das S.K., Mukherjee S. (2012). Biochemical and immunological basis of silymarin effect, a milk thistle (Silybum marianum) against ethanol-induced oxidative damage. Toxicol. Mech. Methods.

[56] Cores Á., Carmona-Zafra N., Clerigué J., Villacampa M., Menéndez J.C. (2023). Quinones as Neuroprotective Agents. Antioxidants.

[57] Ciuclan L., Ehnert S., Ilkavets I., Weng H.L., Gaitantzi H., Tsukamoto H., Ueberham E., Meindl-Beinker N.M., Singer M.V., Breitkopf K. (2010). TGF-beta enhances alcohol dependent hepatocyte damage via down-regulation of alcohol dehydrogenase I. J. Hepatol..

[58] Zhu H.J., Brinda B.J., Chavin K.D., Bernstein H.J., Patrick K.S., Markowitz J.S. (2013). An assessment of pharmacokinetics and antioxidant activity of free silymarin flavonolignans in healthy volunteers: A dose escalation study. Drug Metab. Dispos..

[59] Adachi M., Ishii H. (2002). Role of mitochondria in alcoholic liver injury. Free Radic. Biol. Med..

[60] Bailey S.M., Cunningham C.C. (2002). Contribution of mitochondria to oxidative stress associated with alcoholic liver disease. Free Radic. Biol. Med..

[61] Vidyashankar S., Nandakumar K.S., Patki P.S. (2012). Alcohol depletes coenzyme-Q(10) associated with increased TNF-alpha secretion to induce cytotoxicity in HepG2 cells. Toxicology.

[62] Fernandez-Checa J.C., Kaplowitz N. (2005). Hepatic mitochondrial glutathione: Transport and role in disease and toxicity. Toxicol. Appl. Pharmacol..

[63] Kurose I., Higuchi H., Kato S., Miura S., Ishii H. (1996). Ethanol-induced oxidative stress in the liver. Alcohol. Clin Exp. Res..

[64] Garcia-Ruiz C., Morales A., Colell A., Ballesta A., Rodes J., Kaplowitz N., Fernandez-Checa J.C. (1995). Feeding S-adenosyl-L-methionine attenuates both ethanol-induced depletion of mitochondrial glutathione and mitochondrial dysfunction in periportal and perivenous rat hepatocytes. Hepatology.

[65] Iimuro Y., Bradford B.U., Yamashina S., Rusyn I., Nakagami M., Enomoto N., Kono H., Frey W., Forman D., Brenner D. (2000). The glutathione precursor L-2-oxothiazolidine-4-carboxylic acid protects against liver injury due to chronic enteral ethanol exposure in the rat. Hepatology.

[66] Mari M., Morales A., Colell A., Garcia-Ruiz C., Fernandez-Checa J.C. (2009). Mitochondrial glutathione, a key survival antioxidant. Antioxid. Redox Signal..

[67] Das S.K., Vasudevan D.M. (2007). Alcohol-induced oxidative stress. Life Sci..

[68] Foronjy R., D’Armiento J. (2006). The Effect of Cigarette Smoke-derived Oxidants on the Inflammatory Response of the Lung. Clin. Appl. Immunol. Rev..

[69] Mudway I.S., Kelly F.J., Holgate S.T. (2020). Oxidative stress in air pollution research. Free Radic. Biol. Med..

